# Traditional Chinese medicine use and risk of type 2 diabetes mellitus among patients with prediabetes: a population-based cohort study

**DOI:** 10.1186/s13020-025-01214-x

**Published:** 2025-10-10

**Authors:** Zilin Long, Houyu Zhao, Yueqi Yin, Yexiang Sun, Peng Shen, Hongbo Lin, Junchang Liu, Siyan Zhan, Zhiqin Jiang, Feng Sun

**Affiliations:** 1 Department of Epidemiology and Biostatistics, School of Public Health, Peking University, Beijing, China; 2 Key Laboratory of Epidemiology of Major Diseases (Peking University), Ministry of Education, Beijing, China; 3 Research Center of Clinical Epidemiology, Peking University Third Hospital, Beijing, China; 4 Yinzhou District Center for Disease Control and Prevention, Ningbo, China; 5 Xinjiang Medical University Institute of Traditional Chinese Medicine, Xinjiang, China; 6 Xinjiang Uygur Autonomous Region Academy of Traditional Chinese Medicine, Urumqi Xinjiang, China; 7 School of Medicine, Shihezi University, Shihezi, 832000 China

**Keywords:** Traditional Chinese medicine, Prediabetes, Type 2 diabetes mellitus, Real-world study

## Abstract

**Background:**

Prediabetes was a reversible process in the development of type 2 diabetes mellitus (T2DM). Traditional Chinese medicine (TCM) was used to regulate blood glucose for thousands of years. However, there was a lack of real-world evidence on the long-term impact of TCM in prediabetic populations. This study aimed to evaluate the association between the use of TCM and T2DM incidence among individuals with prediabetes.

**Methods:**

A long-term population-based cohort study of participants with prediabetes was conducted using the Yinzhou Regional Health Care Database (YRHCD). A cox model with propensity score (PS) matching was applied to estimate the hazard ratio (HR) of the association between the use of TCM and T2DM. Various subgroup analyses and multiple sensitivity analyses were also performed to demonstrate the robustness of results.

**Results:**

A total of 14,164 patients with prediabetes were included from the YRHCD between 2009 and 2024, among whom 12,252 participants were TCM users and 1912 initiated western medications (WM). In the primary analysis, the incidence of T2DM was 33.95 and 94.85 per 1000 person-years in users of TCM and WM, respectively. TCM use was associated with a significantly lower risk of developing T2DM (HR 0.32 [95%CI 0.27, 0.38]) after controlling confounding using PS matching. The results were generally consistent in various subgroup analyses and sensitivity analyses.

**Conclusion:**

Use of TCM was associated with a decreased T2DM incidence in patients with prediabetes in the study region.

**Supplementary Information:**

The online version contains supplementary material available at 10.1186/s13020-025-01214-x.

## Background

Prediabetes is a state of abnormal glucose metabolism with blood sugar levels falling between the normal and diabetes, characterized by impaired fasting glucose (IFG) or impaired glucose tolerance (IGT). Its prevalence is increasing with population aging, urbanization and lifestyle transformations. Projections suggest that by 2045, the prevalence of IGT and IFG will increase by 10% and 6.5%, respectively [1]. Multiple researches have indicated that prediabetes is a high-risk stage of transition to type 2 diabetes mellitus (T2DM), with an annual conversion rate ranging from 4 to 19% [2]. However, it is a reversible process in the development of diabetes. Therefore, how to effectively manage the patients with prediabetes is an important content and great challenge to successfully prevent the onset of T2DM.

Lifestyle intervention including weight loss, exercise, and reasonable diet was recommended as a fundamental therapy for prediabetes. Many studies have so far evaluated the effectiveness of lifestyle modification to prevent T2DM in prediabetes population [3, 4]. However, lifestyle modification was very difficult to be applied without help and monitoring from a professional health care practitioner in reality [5]. Antidiabetic medications was also recommended as an alternative intervention for T2DM prevention in Chinese guidelines. Although existing studies have shown that pharmacological intervention can reduce the incidence of diabetes, its effect showed a decreasing trend over time and the duration of effect was limited [6]. Traditional Chinese medicine (TCM) have been proven to displayed anti-diabetes activities, capable of delaying or preventing the T2DM [7–9]. Thus, it was necessary to explore intervention strategies for prediabetes based on the theories of TCM.

For thousands of years, TCM has consistently emphasized a holistic approach to health, emphasizing syndrome differentiation and individualized treatment. It has played an notable role in relieving symptoms and improving quality of life [10, 11]. TCM offers a unique perspective on the pathogenesis and progression for prediabetes, highlighting the importance of Yin-Yang imbalances and the dysfunction of ZangFu, which symbolizes the internal organs and their respective functions [12]. Moreover, fundamental studies have been undertaken to explore the mechanism of TCM in improving glucose metabolism at cellular and molecular levels [13, 14]. Existing evidence-based clinical practices also demonstrated that TCM can significantly enhance glucose control and improve clinical outcomes in patients with prediabetes, effectively reducing the risk of progression to diabetes [9, 15–18]. However, there is a lack of real-world evidence (RWE) on the long-term impact of TCM in prediabetic populations.

RWE is generated through the systematic analysis and processing of real-world data (RWD), which generated during routine healthcare delivery [19]. RWE complements RCT not only because it can overcome limitations of RCTs, including exorbitant costs, small sample size and time-limited follow-up [20], but also due to its ability to capture treatment effects in routine clinical settings and heterogeneous populations [19]. Real-word studies of TCM considers the actual clinical treatment as the exposure factor, thus it contributes to objectively evaluate the clinical efficacy of TCM and endowing research results with external validity, which is favored by scholars of TCM. For instance, researchers in Taiwan have conducted numerous studies on the risk and progression of diabetes utilizing real-world medical data [8, 21–24]. However, the association between TCM and the risk of T2DM in patients with prediabetes has not yet been assessed.

Therefore, we conducted a population-based cohort study to evaluate the association between the use of TCM and T2DM incidence among individuals with prediabetes in mainland China, using data from the Yinzhou Regional Health Care Database (YRHCD).

## Methods

### Data sources

We assembled a retrospective cohort of patients who were newly diagnosed with prediabetes in the YRHCD between January 2009 and December 2024. The YRHCD integrated longitudinal information of electronic medical records, disease registry and management, death registry, and other healthcare services in the Yinzhou District, Ningbo City of China [25, 26]. Longitudinal records of personal demographics, diagnosis descriptions and ICD-10 codes (International Classification of Diseases, 10th revision), drug prescriptions and outpatient and inpatient visits were extracted and linked for information of drug exposure, covariates, and outcome identification.

The study was approved by the ethical review board of Peking University Health Science Center (approval number: IRB00001052-24178). Informed consent was not required owing to the use of anonymized routine data.

## Study subjects

We defined newly diagnosed prediabetes according to ICD-10 code R73.01, R73.02, R73.03, R73.052, and R73.002 with a washout period of 12 months during which no records of diabetes were allowed. Further patients were included if they aged between 18 and 80 years.

An exposure assessment window (EAW) of two years following the date of the first diagnosis of prediabetes was applied for measuring TCM use. Index date was defined as the end of the EAW. By setting a unified exposure window, it was ensured that two groups of research subjects are consistent in terms of the disease course, the exposure duration, and the follow-up period. To better illustrate the cohort design, Figure S1 give a time diagram.

The exposure of TCM during the EAW was defined as any Chinese herbal medicine record or Chinese patent medicine record with hypoglycemic effect (including Shenqi Jiangtang, Jinqi Jiangtang, Xiaoke pill, Jinlida, Jiangtang A tablet, Xiaoke Jiangtang capsule, Jiangtangning capsule, Jiangtangning capsule, etc.). While participants used any routine western antidiabetic medicines in the EAW was consisted of conventional western medicine (WM) group.

Patients were excluded if they meet any of the following criteria: (1) received a diagnosis of any diabetes before the index date; (2) received combination treatment of TCM and conventional western antidiabetic medications in the EAW; (3) had no any prescription record in the EAW; (4) missed information of age and sex; (5) had less than 2 years of continuous records in the YRHCD; (6) died, dropped out, or were diagnosed with any diabetes before the index date.

## Outcome and follow-up

The primary outcome was T2DM defined according to the ICD‐10 codes E11. The date of the first diagnosis of T2DM was defined as the outcome date. Our primary analysis was to emulate an intention-to-treat (ITT) analysis to assess any exposure effects of TCM use on the risk of T2DM. Participants were followed up from the index date until the diagnosis of T2DM, death, last medical record in the database, or the end of the study period (December 31, 2024), whichever occurred first.

## Covariates

Potential confounders were identified according to previous studies [27, 28] and measured before the cohort entry date. These factors included demographic characteristics (age, sex); body mass index (BMI); fasting plasma glucose (FPG); comorbidities, and prescription drugs.

Comorbidities included hypertension, hyperuricemia, hyperlipidemia, cerebrovascular disease (CVD), coronary heart disease (CHD), non-alcoholic fatty liver disease (NAFLD), anxiety and depression, sleep disorders, cancer, chronic pain, and obesity.

Use of prescription drugs including other drugs that may affect blood sugar (steroid drugs, anxiolytic and antidepressant drugs, lipid-lowering drugs, and antihypertensive drugs).

## Statistical analyses

Descriptive statistics were employed to summarize baseline covariates, with continuous variables presented as mean and standard deviation, and categorical variables summarized by frequency and percentage. Standardized mean difference (SMD) was used to evaluate the balance of covariates between the TCM group and the CWM group. A SMD less than 0.1 was considered a comparable balance in the covariates [29].

Propensity score matching (PSM) was applied for controlling baseline confounding and a Cox regression model was used to estimate the hazard ratios (HR) with 95% confidence interval (CI) for the association between TCM and T2DM. All analyses were conducted using R4.3.2 software, at a *P* < 0.05 statistically significant level. The PSM model used all measured covariates listed above as independent variables, and a logistic regression model was used to estimate the propensity score. The 1:1 nearest neighbor matching method with was used for matching, and the caliper value was 0.01 times of the standard deviation of the logit of the propensity score.

## Subgroup analyses

We further assessed the risk of T2DM in TCM users within different subgroups for checking potential interactions. These subgroup analyses were conducted based on participants' sex, age (< 45 and ≥ 45 years), BMI (≥ 28, 24–27.9 and, < 24 kg/m^2^), FBG levels (6.5–6.9 and < 6.5 mmol/L), risk factors, comorbidities, and co-use of prescription drugs. PSM were re-estimated separately for each subgroup.

## Sensitivity analyses

We conducted multiple sensitivity analyses to examine the robustness of the results.

Sensitivity analyses mainly focused on six aspects, including analysis method, imputation of missing data, diagnostic criteria, competing risk by all-cause mortality, exposure classification and study population.

First, two different analysis methods were adopted to assess the effect of TCM on the occurrence of T2DM among individuals with prediabetes, namely multivariable Cox regression analysis in the unmatched full population data set and Cox regression analysis following inverse probability of treatment weighting (IPTW).

Second, multiple interpolation was used to fill the missing BMI and FPG values and PSM were re-estimated on the post-imputation data set.

Third, we use an alternative definition of prediabetes participants who had at least two prediabetes diagnoses within 1 year of a new prediabetes diagnosis.

Fourth, the Fine-Gray subdistribution hazard model was employed to check possible competing risk by death from any cause.

Fifth, three alternative TCM definition was applied: (1) TCM definition 1: We restricted the TCM users to patients of whom the date of TCM prescription all fell within 1–3 days of the prediabetes diagnosis date. This was to ensure that TCM users seek TCM treatments because of prediabetes. (2) TCM definition 2: According to the TCM pathogenesis of prediabetes and the therapeutic effects of Chinese herbal medicines, TCM was considered for prediabetes only if the prescription contained at least one of the following categories of Chinese herbals: heat-clearing drugs, blood-activating drugs, moisture-clearing drugs, dampness-clearing drugs, digestion drugs, or tonifying drugs. (3) TCM definition 3: TCM users were defined as participants meeting both of the above two criteria.

Sixth, two different study populations were defined. First, for the study population 2, participants were required to had at least two definitive diagnoses of prediabetes within 1 year of a new prediabetes diagnosis and the date of each prescription had to be within 1–3 days of prediabetes diagnosis date during the EAW. Second, for the study population 3: participants were required to meet all of the three criteria: (1) had ≥ 2 records of prediabetes diagnoses within 1 year of a new prediabetes diagnosis; (2) date of each prescription had to be within 1–3 days of each diagnosis date; (3) TCM prescriptions had to include at least one of the following categories of Chinese herbals: heat-clearing drugs, blood-activating drugs, moisture-clearing drugs, dampness-clearing drugs, digestion drugs and tonifying drugs.

## Post hoc analyses

We analysed frequencies of different herbs to understand the prescription patterns in prediabetes patients. Effects of the top 10 most commonly prescribed herbal medicines were assessed separately, with participants using each kind of herbs being compared with individuals who did not use the top 10 Chinese herbs. Cox regression was applied to assess the hazard ratio. These analyses were explanatory and we did not adjust for multiple testing.

## Results

### Demographic characteristics

**Fig.1:**
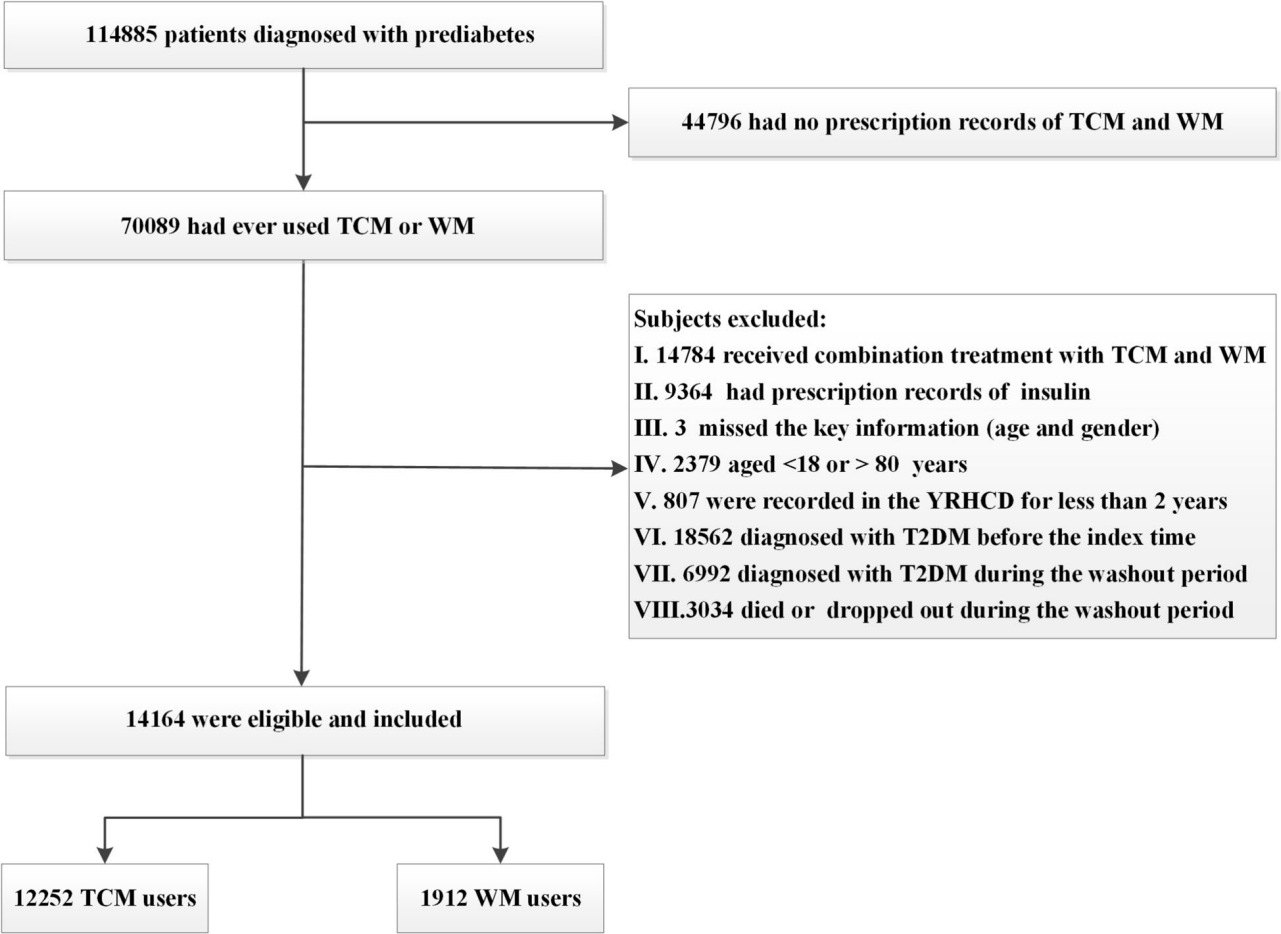
Flowchart of participants in the study cohort. *TCM* Traditional Chinese medicine, *WM* Western medicine, *YRHCD* Yinzhou Regional Health Care Database *T2DM* Type 2 diabetes

A total of 114 885 individuals diagnosed with prediabetes were identified in the YRHCD between 2009 and 2024. 14 164 patients (12 252 TCM users and 1 912 WM users) were eligible according the inclusion and exclusion criteria and were enrolled in this study (Fig. 1), with a median follow-up time of 4.1 (IQR, 2.0 ~ 5.8; maximum 13.8) and 4.0 (IQR, 1.15 ~ 5.38; maximum 13.6) years, respectively. The mean age was 57.44 ± 11.98 years and 66.07% (9 358) of participants were female. WM users had higher BMI compared with TCM users (Table 1). Also, there were higher proportions of participants who co-used steroid drugs and lipid-lowering drugs in TCM users at baseline. However, all baseline characteristics were effectively balanced between groups of TCM and WM users after propensity score matching (SMD < 0.1 for all covariates).

**Table 1:** Baseline covariates of prediabetes patients between TCM group and WM group

	Unmatched			PSM		
	TCM (n = 12,252)	WM (n = 1912)	SMD	TCM (n = 1912)	WM (n = 1912)	SMD
Age, years	57.96 (11.56)	54.08 (13.94)	0.303	54.16 (13.12)	54.08 (13.94)	0.006
Age ≥ 45 years	10,756 (87.8)	1448 (75.7)	0.316	1454 (76.0)	1448 (75.7)	0.007
Female	8399 (68.6)	959 (50.2)	0.381	928 (48.5)	959 (50.2)	0.032
BMI	23.95 (3.26)	24.44 (3.59)	0.143	24.36 (3.56)	24.44 (3.59)	
BMI categories						
≥ 28 kg/m^2^	1124 (9.2)	208 (10.9)	0.455	193 (10.1)	208 (10.9)	0.055
24 kg/m^2^ ≤ BMI < 28 kg/m^2^	4055 (33.1)	518 (27.1)		553 (28.9)	518 (27.1)	
< 24 kg/m^2^	5894 (48.1)	692 (36.2)		704 (36.8)	692 (36.2)	
Unknown	1179 (9.6)	494 (25.8)		462 (24.2)	494 (25.8)	
FPG, mmol/l	6.24 (0.24)	6.27 (0.26)	0.126	6.29 (0.78)	6.27 (0.26)	0.064
Comorbidity						
Hypertension	7862 (64.2)	1153 (60.3)	0.080	1153 (60.3)	1153 (60.3)	< 0.001
Hyperuricemia	819 (6.7)	82 (4.3)	0.105	66 (3.5)	82 (4.3)	0.043
Hyperlipidemia	6699 (54.7)	688 (36.0)	0.382	656 (34.3)	688 (36.0)	0.035
Comorbidity						
CVD	4890 (39.9)	406 (21.2)	0.414	404 (21.1)	406 (21.2)	0.003
CHD	3947 (32.2)	379 (19.8)	0.285	362 (18.9)	379 (19.8)	0.022
NAFLD	1039 (8.5)	95 (5.0)	0.141	96 (5.0)	95 (5.0)	0.002
Anxiety/depression	2470 (20.2)	160 (8.4)	0.342	162 (8.5)	160 (8.4)	0.004
Sleep disorders	6838(55.8)	525 (27.5)	0.601	537 (28.1)	525 (27.5)	0.014
Cancer	706 (5.8)	48 (2.5)	0.164	39 (2.0)	48 (2.5)	0.032
Chronic pain	4779 (39.0)	392 (20.5)	0.413	380 (19.9)	392 (20.5)	0.016
Obesity	1167 (9.5)	228 (11.9)	0.078	210 (11.0)	228 (11.9)	0.030
Other comorbidity	382 (3.1)	89 (4.7)	0.080	70 (3.7)	89 (4.7)	0.050
Medication						
Steroid drugs	5311 (43.3)	546 (28.6)	0.312	573 (30)	546 (28.6)	0.031
Anxiolytic/antidepressant drugs	676(5.5)	69 (3.6)	0.092	69 (3.3)	69 (3.6)	0.017
Lipid-lowering drugs	4824 (39.4)	603 (31.5)	0.164	566 (29.6)	603 (31.5)	0.042
Antihypertensive drugs	7107 (58.0)	1124 (58.8)	0.016	1065 (55.7)	1124 (58.8)	0.062

*TCM* Traditional Chinese medicine, *WM* Western medicine, *PSM* Propensity score matching, *IPTW* Inverse probability of treatment weighting, *BMI* Body mass index, *CVD* Cerebrovascular disease, *CHD* Coronary heart disease, *NAFLD* Non-alcoholic fatty liver disease, *SMD* Standardized mean difference

## Primary and subgroup analyses

**Fig.2:**
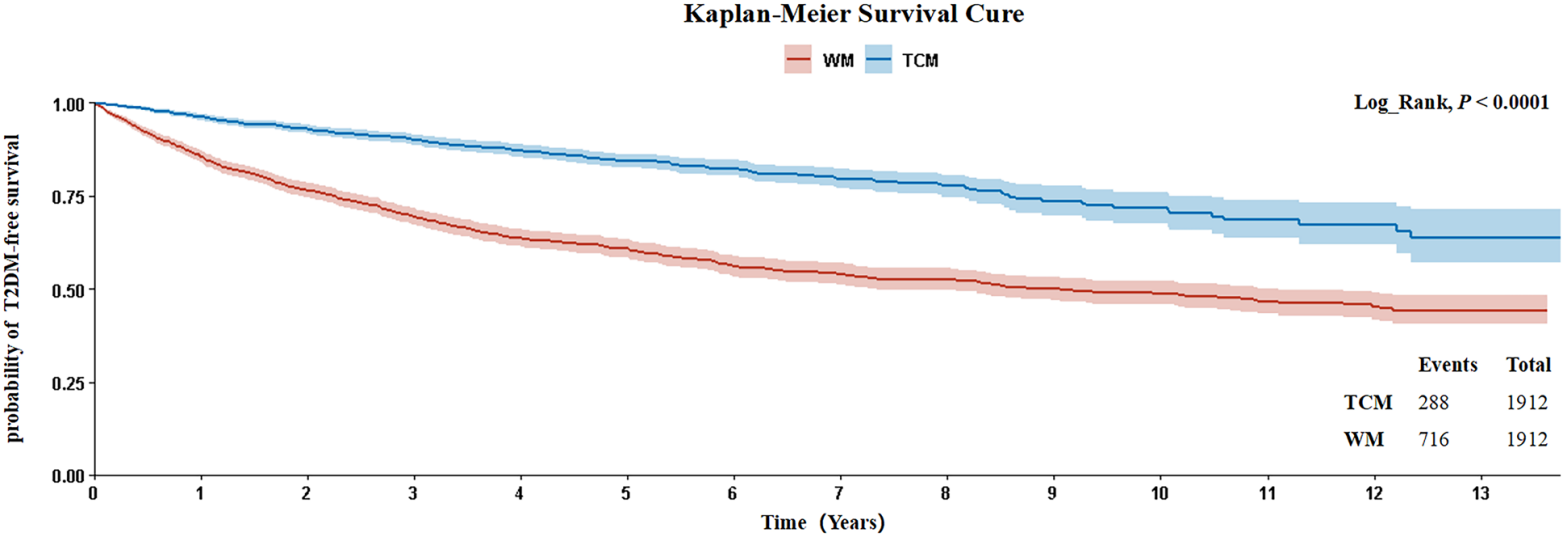
Survival curves of the users of TCM and WM. *TCM* Traditional Chinese medicine, *WM* Western medicine *T2DM* Type 2 diabetes

A total of 1 002 T2DM cases occurred among PSM participants, including 286 TCM users and 716 WM users. The incidence of T2DM was 33.95 per 1000 person-years for TCM users and 94.85 per 1000 person-years for WM users. Compared with WM users, TCM users were at significantly lower risk of developing T2DM (HR 0.32 [95%CI 0.27, 0.38]). The survival curve of T2DM between TCM and WM users demonstrated the protective effect of TCM usage over a maximum of 13 years of follow-up (p < 0.001) (Fig. 2).

**Fig.3:**
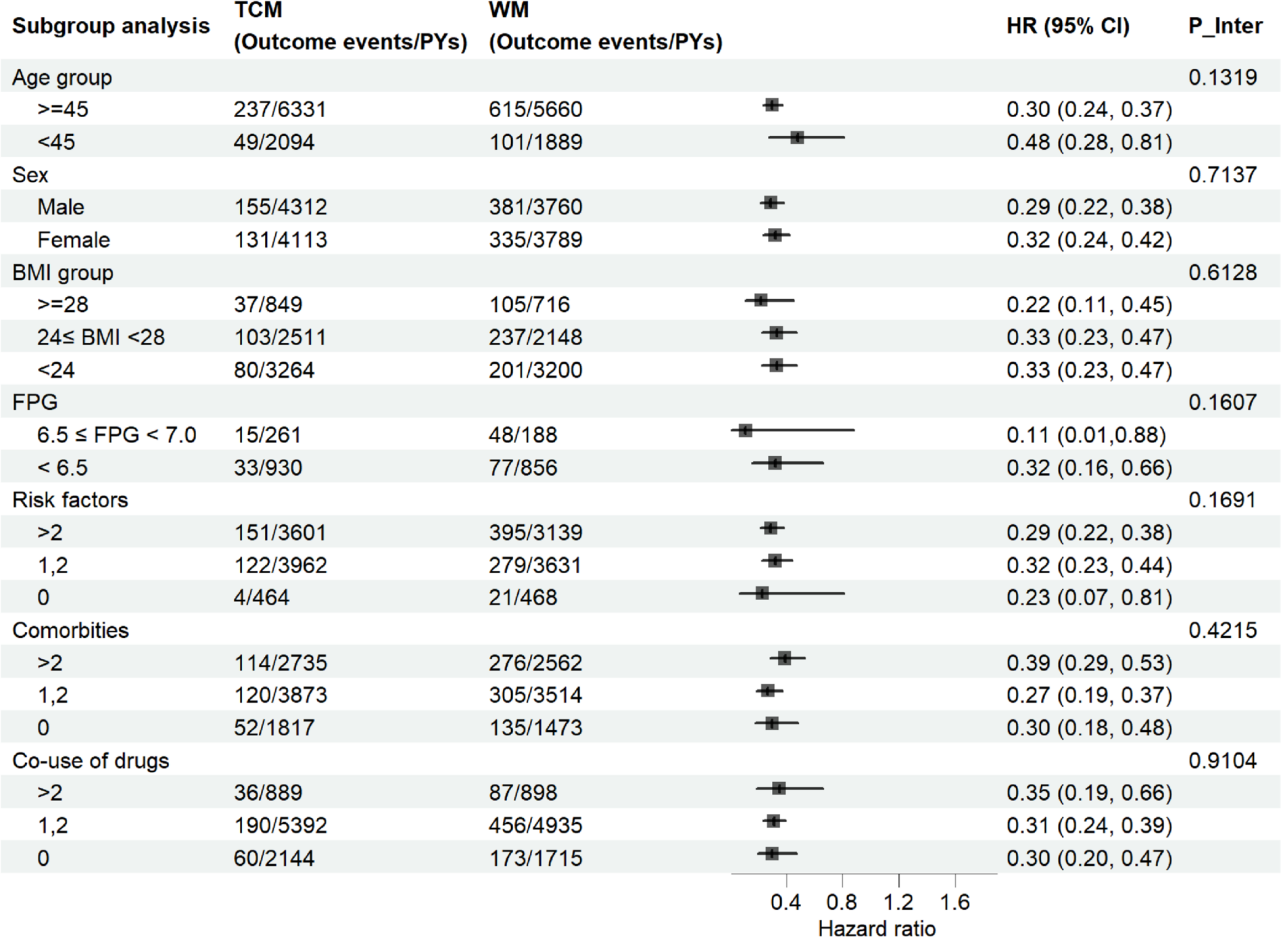
Results of subgroup analyses. *TCM* Traditional Chinese medicine, *WM* Western medicine, *BMI* Body mass index, *FPG* Fasting plasma glucose, *PYs* Person-years, *HR* Hazard ratio, *P Inter P* value for interaction. ^a^Subgroup analyses were performed only in the nonmissing data group

Subgroup analyses revealed significant inverse association between use of TCM and incidence of T2DM in different subpopulations of prediabetes patients. There was no significant interaction in all subgroup analyses (Fig. 3).

## Sensitivity analysis

**Fig.4:**
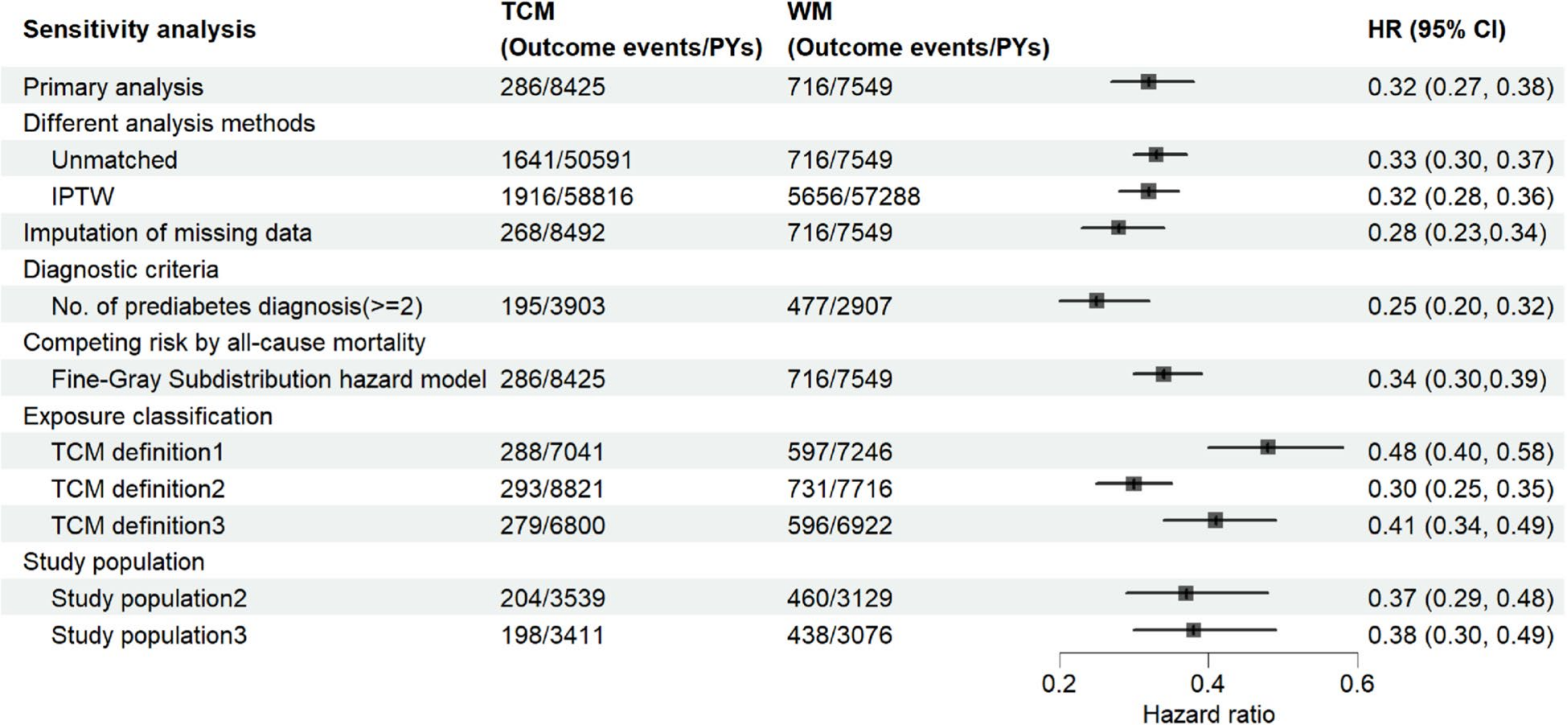
Results of sensitivity analyses. *TCM* Traditional Chinese medicine *WM* Western medicine, *PYs* Person-years, *HR* Hazard ratio, *PSM* Propensity score matching, *IPTW* Inverse probability of treatment weighting

Figure 4 presented the results of all sensitivity analyses and demonstrated that TCM.

Users exhibited a significantly reduction in the risk of T2DM compared with WM users.

First, cox regression analysis in the unmatched full population data set showed that TCM users was associated with a 67% reduction in the incidence of T2DM (HR 0.33 [95% CI 0.30, 0.37]). The IPTW model exhibited a similar protective effect of TCM users with an HR of 0.32 (95% CI 0.28–0.36). Supplementary Figure S2 and Figure S3 gave the Kaplan–Meier survival curves in patients with prediabetes.

The missing data proportion for BMI was approximately 11.2%, while that for FPG exceeded 30%. We incorporated missing values as a separate category in the primary analysis. Although this approach helps preserve sample size to some extent, it may also introduce bias. Therefore, to further evaluate the impact of different missing data handling methods on the study outcomes, we conducted a sensitivity analysis using multiple imputation for the missing values of BMI and FPG. After multiple imputation of missing data and 1:1 propensity score matching, the risk of T2DM in the TCM group was significantly lower than that in the WM group (HR 0.28 [95% CI 0.23, 0.34]).

Third, a total of 5987 patients with at least two prediabetes diagnoses were included in the study, of whom 906 were WM users and 5,081 were TCM users. 906 eligible patients in each groups were selected after 1:1 PSM. We found that TCM users showed a lower incidence of T2DM (HR 0.25 [95% CI 0.20, 0.32]). Fourth, competing risk due to all-cause death did not indicate a substantial impact on the results, with an HR of 0.34 (95% CI 0.30–0.39).

Fifth, there were 1669 TCM users meeting the TCM definition 1. After 1:1 propensity score matching, our analysis presented that TCM users was associated with a 52% reduction in the risk of T2DM (HR 0.48 [95% CI 0.40, 0.58]). A total of 11,597 TCM users met the TCM definition 2 and significantly reduced the incidence rate of T2DM with an HR of 0.30 (95% CI 0.25–0.35). Further, 1606 TCM users were included when using the TCM definition 3 and TCM presented a statistically significant reduction in T2DM risk (HR 0.41 [95% CI 0.34, 0.49]).

Sixth, after limiting the frequency of prediabetes diagnosis and the relationship between prescription and diagnosis, 2322 eligible patients (877 TCM users and 1 445 WM users) were included. The incidence of T2DM was 57.64 per 1000 person-year for TCM users and 147.01 per 1 000 person-year for WM users in the PSM model (HR 0.37 [95% CI 0.29, 0.48]). Additionally, 2249 participants were eligible for selection 2, including 843 TCM users and 1 451 WM users. TCM group still showed a similar protective effect in reducing the risk of T2DM (HR 0.38 [95% CI 0.30, 0.49]).

## Most commonly used herbs and their association with T2DM risk

The top 10 most frequently individual herbs for prediabetes patients were Gan-Cao (*Glycyrrhiza uralensis *Fisch), Fu-Ling (*Poria*), Bai-Zhu (*Atractylodes macrocephala *Koidz), Bai-Shao (*Paeoniae radix alba*), Chen-Pi (*Aurantii nobillis pericarpium*), Dang-Gui (*Angelicae radix*), Chai-Hu (*Bupleuri radix*), Dang-Shen (*radix codonopsis*), Ban-Xia (*Pinelliae rhizoma*) and Huang-Qin (*Scutellariae radix*).

Cox proportional hazards regression analysis showed that the use of top 10 herbs was significantly associated with lower risk of T2DM (Table 2). However, the most commonly used herbs ananlyses were post-hoc and exploratory.

**Table 2:** T2DM risk associated the 10 most commonly used Chinese herbs for patients with predibetes

Chinese herbs	No. of user	Outcome events /PYs	HR (95% CI)^a^	HR (95% CI)^b^
Gan-Cao (*Glycyrrhiza uralensis *Fisch)	8078	1068/33461	0.60 (0.55, 0.65)	0.55 (0.50, 0.62)
Fu-Ling (*Poria*)	7052	907/29469	0.65 (0.60, 0.71)	0.56 (0.51, 0.63)
Bai-Zhu (*Atractylodes macrocephala *Koidz)	6212	832/25654	0.74 (0.68, 0.81)	0.73 (0.66, 0.82)
Bai-Shao (*Paeoniae radix alba*)	5405	702/22921	0.71 (0.65, 0.78)	0.74 (0.65, 0.83)
Chen-Pi (*Aurantii nobillis pericarpium*)	5121	700/20817	0.81 (0.74, 0.88)	0.82 (0.73, 0.93)
Dang-Gui (*Angelicae radix*)	5196	697/22046	0.76 (0.69, 0.83)	0.78 (0.69, 0.88)
Chai-Hu (*Bupleuri radix*)	4403	559/18245	0.74 (0.67, 0.81)	0.77 (0.67, 0.88)
Dang-Shen (*radix codonopsis*)	4715	670/19932	0.82 (0.75, 0.90)	0.79 (0.70, 0.90)
Ban-Xia (*Pinelliae rhizoma*)	4186	529/16820	0.75 (0.68, 0.83)	0.73 (0.63, 0.83)
Huang-Qin (*Scutellariae radix*)	4059	534/16777	0.75 (0.68, 0.83)	0.86 (0.75, 0.99)

HR (95% CI)^a^: Hazard ratio was analyzed by cox regression in the unmatched full population data set

HR (95% CI)^b^: Hazard ratio was analyzed by cox regression of propensity-score-matched participants

## Discussion

In this long-term population-based cohort study, we provided evidence of the association between TCM use and T2DM risk in patients with prediabetes. Our results revealed that patients treated with TCM after the diagnosis of prediabetes showed a significantly lower risk of T2DM compared with WM users (HR 0.32 [95%CI 0.27, 0.38]). Further, our results remained consistent in various subgroup analyses and demonstrated robustness in multiple sensitivity analyses.

Current population-based studies on the association between TCM and the risk of T2DM are primarily from Taiwan, China, and their findings are generally consistent with our study, reporting that TCM has a significant preventive effect on T2DM [21, 30]. However, these studies either did not focus on the prediabetic population or had relatively small sample sizes (with fewer than 400 participants per group). We included 14,164 patients with prediabetes, employed PSM method to adjust for multiple known risk factors and ensured a consistent duration of prediabetes for patients. These measures collectively helped to mitigate indication bias and immortal-time bias to some extent. Nonetheless, given the inherent limitations of real-world studies and the complexity of observational researches, there might still be unidentified residual confounders impacting our findings. These factors might include patients' health awareness, treatment compliance, lifestyle, genetic factors and biological mechanisms. Therefore, the results should be interpreted with caution and further validated in subsequent studies.

An additional contribution of our study is the list of Chinese herbal products that were found to be beneficial in reducing T2DM risk. For instance, Fuling (*Poria*) was one of the most frequently prescribed herbs used to control glucose level for many years and had been confirmed to significantly reduce FPG, 2-h postprandial blood glucose and haemoglobin A1c [31]. Our study still presented that Fuling can lower the T2DM risk by 44% in prediabetes (HR 0.56 [95% CI 0.51, 0.62]). Poria polysaccharide, the main component of Poria, was found to have hypoglycaemic functions. It was demonstrated to improve hyperglycemia through the modulation of gut microbiota and intestinal metabolites [32–34]. Nevertheless, the analyses of these ten commonly prescribed Chinese berbals were post-hoc and exploratory. It should be interpreted with caution and requires validation in prospective studies.

There were some advantages in our study. First, to our knowledge, this was the first study to evaluate the association between the use of TCM and T2DM incidence among individuals with prediabetes in mainland China, using a large cross-regional medical database. Second, the active-comparator design was used in this study, which avoided the risk of time-related biases such as immortal-time bias and lag time bias [35]. Third, our results illustrate long-term effects of TCM, with the longest follow-up period exceeding 13 years. Moreover, multiple subgroup analyses and a series of sensitivity analyses targeting potential risks of bias were also performed, with consistent results demonstrating robustness of our findings.

However, there were limitations in our study. First, despite the fact that the database we used had been integrated across healthcare settings and included data from birth to death, it still lacked information on clinical risk scores (e.g., the Chinese Diabetes Risk Score), waist circumference, abdominal circumference, and patient lifestyle factors (e.g., smoking, alcohol consumption, dietary habits and exercise habits). Dosage information of drugs in the database was also incomplete, making it difficult to study the relationship between cumulative TCM exposure and T2DM incidence. In the future, we should strengthen the construction of real-world healthcare big data and improve the relevant information. Second, our study was not able to evaluate any herbal or other treatments that might have been purchased outside hospitals. Third, our study only represented the Chinese population, and race may influence the transition from prediabetes to T2DM. In the future, cross-regional scientific research cooperation can be sought to realize multi-center and transnational big data research.

Fifth, patients were not randomly divided into TCM users and WM users. Residual confounding may still exist despite a series of adjustments using epidemiological research methods.

## Conclusions

Patients with prediabetes who received TCM therapy appeared to have a nearly 68% risk of developing T2DM compared with WM. The top ten most commonly used Chinese herbs in prediabetes patients all showed a significantly effect in reducing the incidence of T2DM. These findings provided real-world evidence for the effective management of people with prediabetes by TCM. Future prospective randomized clinical trials should be conducted to verify the reliability of our findings.

## Supplementary Information

Supplementary Material 1.

## Data Availability

The data can be provided from corresponding authors upon reasonable request. The original contributions presented in the study were included in the article/ supplementary material, further inquiries can be directed to the corresponding authors.

## Abbreviations

BMI: Body mass index
CHD: Coronary heart disease
CVD: Cerebrovascular disease
FPG: Fasting plasma glucose
HR: Hazard ratio
ICD-10 codes: International classification of diseases, 10th revision
IPTW: Inverse probability of treatment weighting
ITT: Intention-to-treat
NAFLD: Non-alcoholic fatty liver disease
T2DM: Type 2 diabetes
TCM: Traditional Chinese medicine
P_Inter: *P* Value for interaction.
PSM: Propensity score matching
PYs: Person-years
WM: Western medicine
YRHCD: Yinzhou regional health care database

## References

[1] Rooney MR, Fang M, Ogurtsova K, et al. Global prevalence of prediabetes. Diabetes Care. 2023;46(7):1388–94. 10.2337/dc22-2376. 37196350 10.2337/dc22-2376 PMC10442190

[2] Bansal N. Prediabetes diagnosis and treatment: a review. World J Diabetes. 2015;6(2):296–303. 10.4239/wjd.v6.i2.296. 25789110 10.4239/wjd.v6.i2.296 PMC4360422

[3] Gong Q, Zhang P, Wang J, et al. Morbidity and mortality after lifestyle intervention for people with impaired glucose tolerance: 30-year results of the Da Qing Diabetes Prevention Outcome Study. Lancet Diabetes Endocrinol. 2019;7(6):452–61. 10.1016/s2213-8587(19)30093-2. 31036503 10.1016/S2213-8587(19)30093-2 PMC8172050

[4] Galaviz KI, Weber MB, Suvada KB, et al. Interventions for reversing prediabetes: a systematic review and meta-analysis. Am J Prev Med. 2022;62(4):614–25. 10.1016/j.amepre.2021.10.020. 35151523 10.1016/j.amepre.2021.10.020 PMC10420389

[5] Guidelines on the management and prevention of prediabetes. Acta Med Indones. 2014;46(4):348–59. 25633555

[6] Haw JS, Galaviz KI, Straus AN, et al. Long-term sustainability of diabetes prevention approaches: a systematic review and meta-analysis of randomized clinical trials. JAMA Intern Med. 2017;177(12):1808–17. 10.1001/jamainternmed.2017.6040. 29114778 10.1001/jamainternmed.2017.6040 PMC5820728

[7] Wei B, Gao T, Li M, Tian X, Wang J. A real-world observational study on the effect of Qingre Lishi decoction on glycemic profile using continuous glucose monitoring in obese type 2 diabetes adults. Front Endocrinol. 2024;15:1372593. 10.3389/fendo.2024.1372593. 10.3389/fendo.2024.1372593 PMC11300197 39109082

[8] Tsai YY, Chen KJ, Yang YH, Lin YH. Use of traditional Chinese medicine may delay the need for insulin treatment in patients with type 2 diabetes: a population-based cohort study. J Altern Complement Med. 2020;26(7):628–35. 10.1089/acm.2019.0375. 32543210 10.1089/acm.2019.0375

[9] Song Y, Wang H, Qin L, et al. Efficiency and Safety of Chinese herbal medicine in the treatment of prediabetes: a systemic review and meta-analysis of randomized controlled trials. Evid Based Complement Alternat Med. 2020;2020:3628036. 10.1155/2020/3628036. 33123206 10.1155/2020/3628036 PMC7584953

[10] Kang X, Jin D, Ji H, et al. The clinical efficacy of Gegen Qinlian decoction in treating type 2 diabetes is positively correlated with the dose of Coptidis rhizoma: three randomized, doubleblind, dose-parallel controlled clinical trials. Drug Des Devel Ther. 2024;18:5573–82. 10.2147/dddt.S487315. 39650851 10.2147/DDDT.S487315 PMC11625183

[11] Tian J, Jin D, Bao Q, et al. Evidence and potential mechanisms of traditional Chinese medicine for the treatment of type 2 diabetes: a systematic review and meta-analysis. Diabetes Obes Metab. 2019;21(8):1801–16. 10.1111/dom.13760. 31050124 10.1111/dom.13760

[12] Li Z, Xu C. The fundamental theory of traditional Chinese medicine and the consideration in its research strategy. Front Med. 2011;5(2):208–11. 10.1007/s11684-011-0126-x. 21695627 10.1007/s11684-011-0126-x

[13] Wang C, An T, Lu C, et al. Tangzhiping decoction improves glucose and lipid metabolism and exerts protective effects against white adipose tissue dysfunction in prediabetic mice. Drug Des Devel Ther. 2024;18:2951–69. 10.2147/dddt.S462603. 39050798 10.2147/DDDT.S462603 PMC11268521

[14] Xie Y, Li Z, Fan Y, et al. Integrated gut microbiome and UHPLC-MS metabolomics to reveal the prevention mechanism of pidanjiangtang granules on IGT Rats. Phytomedicine. 2024;135:156201. 10.1016/j.phymed.2024.156201. 39531936 10.1016/j.phymed.2024.156201

[15] Ji H, Zhao X, Chen X, et al. Jinlida for diabetes prevention in impaired glucose tolerance and multiple metabolic abnormalities: the FOCUS randomized clinical trial. JAMA Intern Med. 2024;184(7):727–35. 10.1001/jamainternmed.2024.1190. 38829648 10.1001/jamainternmed.2024.1190 PMC11148787

[16] Gao J, Shi J, Ma X, et al. Effects of ginseng berry saponins from *panax ginseng* on glucose metabolism of patients with prediabetes: a randomized, double-blinded, placebo-controlled, crossover trial. Phytomedicine. 2024;132:155842. 10.1016/j.phymed.2024.155842. 39004031 10.1016/j.phymed.2024.155842

[17] Fang Z, Bi Z, Zhao J, et al. The effects of Danzhi Jiangtang capsule on clinical indices and vascular endothelial function in patients with impaired glucose tolerance of Qi-Yin deficiency type. Ann Med. 2023;55(2):2291185. 10.1080/07853890.2023.2291185. 38146741 10.1080/07853890.2023.2291185 PMC10763911

[18] Ma K, Tian C, Guo C, Li M. The efficacy of Chinese patent medicine intervention on blood glucose and lipid in prediabetes: a meta-analysis. Heliyon. 2022;8(12):e12112. 10.1016/j.heliyon.2022.e12112. 36544847 10.1016/j.heliyon.2022.e12112 PMC9761722

[19] Dang A. Real-world evidence: a primer. Pharmaceut Med. 2023;37(1):25–36. 10.1007/s40290-022-00456-6. 36604368 10.1007/s40290-022-00456-6 PMC9815890

[20] Fitzke H, Fayzan T, Watkins J, Galimov E, Pierce BF. Real-world evidence: state-of-the-art and future perspectives. J Comp Eff Res. 2025. 10.57264/cer-2024-0130. 40051332 10.57264/cer-2024-0130 PMC11963347

[21] Liao W-T, Su C-C, Lee M-T, et al. Integrative Chinese herbal medicine therapy reduced the risk of type 2 diabetes mellitus in patients with polycystic ovary syndrome: a nationwide matched cohort study. J Ethnopharmacol. 2019;243:112091. 10.1016/j.jep.2019.112091. 31325604 10.1016/j.jep.2019.112091

[22] Zheng Y, Yang F, Han L, et al. Efficacy of Chinese herbal medicine in the treatment of moderate-severe painful diabetic peripheral neuropathy: a retrospective study. J Diabetes Res. 2019;2019:4035861. 10.1155/2019/4035861. 31950063 10.1155/2019/4035861 PMC6948321

[23] Lu HL, Su YC, Lin MC, Sun MF, Huang ST. Integrating Chinese and Western medicines reduced the incidence of hepatocellular carcinoma in patients with diabetes mellitus: a Taiwanese population-based cohort study. Complement Ther Med. 2020;49:102332. 10.1016/j.ctim.2020.102332. 32147062 10.1016/j.ctim.2020.102332

[24] Chan KW, Chow TY, Yu KY, et al. Effectiveness of integrative Chinese-Western medicine for chronic kidney disease and diabetes: a retrospective cohort study. Am J Chin Med. 2022;50(2):371–88. 10.1142/s0192415x2250015x. 35168474 10.1142/S0192415X2250015X

[25] Zhao H, Zhuo L, Sun Y, Shen P, Lin H, Zhan S. Thiazolidinedione use is associated with reduced risk of dementia in patients with type 2 diabetes mellitus: a retrospective cohort study. J Diabetes. 2023;15(2):97–109. 10.1111/1753-0407.13352. 36660897 10.1111/1753-0407.13352 PMC9934955

[26] Zhuo L, Zhang B, Yin Y, et al. Use of sodium-glucose cotransporter-2 inhibitors and risk of dementia: a population-based cohort study. Diabetes Obes Metab. 2025. 10.1111/dom.16239. 39927408 10.1111/dom.16239

[27] Lundgrin EL, Hatipoglu B. Trending modalities in type 2 diabetes prevention. J Clin Endocrinol Metab. 2025;110(Supplement_2):S187–92. 10.1210/clinem/dgaf040. 39998920 10.1210/clinem/dgaf040

[28] LeBlanc ES, Smith N, Hwang D, et al. The sleep for health study: a randomized clinical trial of the impact of insomnia treatment on glycemia in people with prediabetes. Contemp Clin Trial. 2025;149:107796. 10.1016/j.cct.2024.107796. 10.1016/j.cct.2024.107796 PMC11788072 39730078

[29] Austin PC, Stuart EA. Moving towards best practice when using inverse probability of treatment weighting (IPTW) using the propensity score to estimate causal treatment effects in observational studies. Stat Med. 2015;34(28):3661–79. 10.1002/sim.6607. 26238958 10.1002/sim.6607 PMC4626409

[30] Weng SW, Chang CC, Chen TL, et al. Risk of diabetes in stroke patients who used Bu Yang Huan Wu Tang: a nationwide propensity-score matched study. Phytomedicine. 2021;80:153376. 10.1016/j.phymed.2020.153376. 33086171 10.1016/j.phymed.2020.153376

[31] Di YM, Sun L, Lu C, et al. Benefits of herbal formulae containing *Poria cocos* (Fuling) for type 2 diabetes mellitus: a systematic review and meta-analysis. PLoS ONE. 2022;17(12):e0278536. 10.1371/journal.pone.0278536. 36455062 10.1371/journal.pone.0278536 PMC9714931

[32] Sun SS, Wang K, Ma K, Bao L, Liu HW. An insoluble polysaccharide from the sclerotium of *Poria cocos* improves hyperglycemia, hyperlipidemia and hepatic steatosis in ob/ob mice via modulation of gut microbiota. Chin J Nat Med. 2019;17(1):3–14. 10.1016/s1875-5364(19)30003-2. 30704621 10.1016/S1875-5364(19)30003-2

[33] Zhu L, Ye C, Hu B, et al. Regulation of gut microbiota and intestinal metabolites by *Poria cocos* oligosaccharides improves glycolipid metabolism disturbance in high-fat diet-fed mice. J Nutr Biochem. 2022;107:109019. 10.1016/j.jnutbio.2022.109019. 35472435 10.1016/j.jnutbio.2022.109019

[34] Xie Q, Jia X, Zhang W, et al. Effects of *Poria cocos* extract and protein powder mixture on glucolipid metabolism and rhythm changes in obese mice. Food Sci Nutr. 2023;11(5):2356–71. 10.1002/fsn3.3245. 37181308 10.1002/fsn3.3245 PMC10171496

[35] Suissa S, Dell’Aniello S. Time-related biases in pharmacoepidemiology. Pharmacoepidemiol Drug Saf. 2020;29(9):1101–10. 10.1002/pds.5083. 32783283 10.1002/pds.5083

