# Supplementary material for: Traditional Chinese medicine use and risk of type 2 diabetes mellitus among patients with prediabetes: a population-based cohort study
Source: Chin Med. 2025 Oct 10;20:171. doi: 10.1186/s13020-025-01214-x (PMC12513027; doi:10.1186/s13020-025-01214-x)
Supplement: Supplementary file 1 — Supplementary Material 1. [file 13020_2025_1214_MOESM1_ESM.docx]

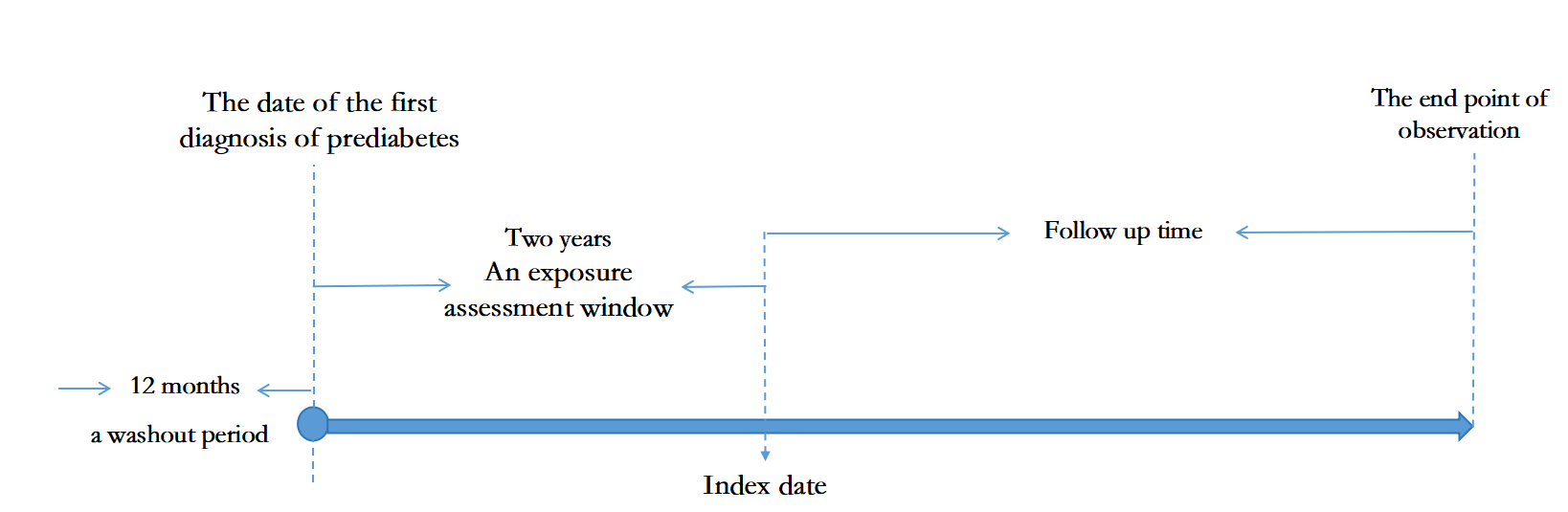


**Figure S1** a time diagram of the cohort.


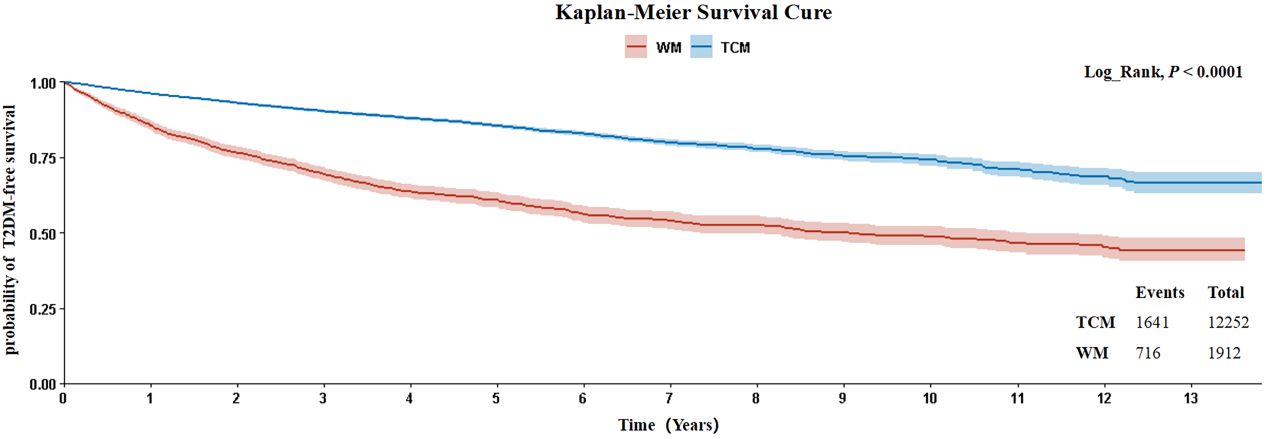


**Figure S2** Survival curves of the users of TCM and WM in the unmatched full population data set. TCM, traditional Chinese medicine; WM, western medicine; T2DM, type 2 diabetes.


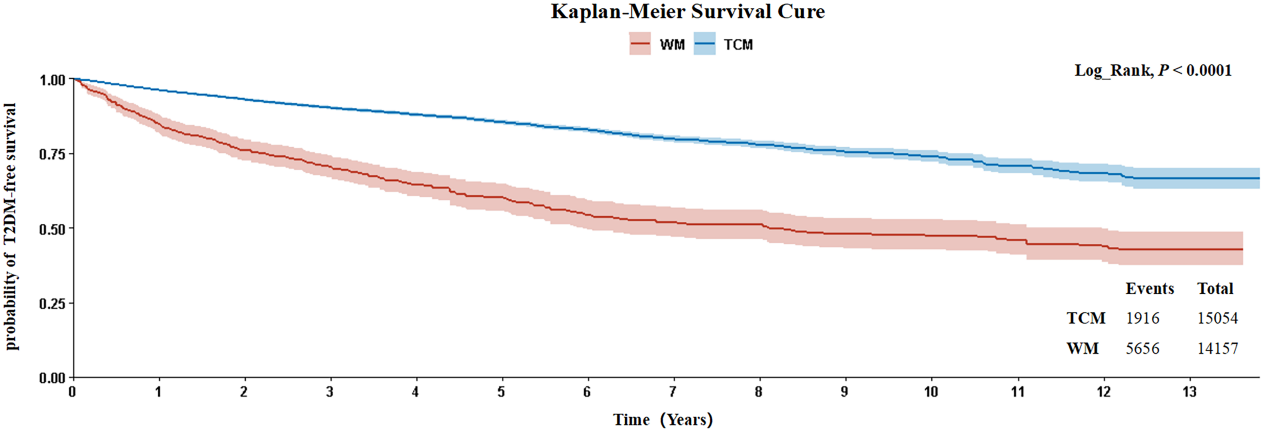


**Figure S3** Survival curves of the users of TCM and WM following inverse probability of treatment weighting. TCM, traditional Chinese medicine; WM, western medicine; T2DM, type 2 diabetes.
